# Microstructural Evolution and Mechanical Performance of Two Joints of Medium-Mn Stainless Steel with Low- and High-Alloyed Steels

**DOI:** 10.3390/ma16041624

**Published:** 2023-02-15

**Authors:** Mahmoud Khedr, I. Reda Ibrahim, Matias Jaskari, Mohammed Ali, Hamed A. Abdel-Aleem, Tamer S. Mahmoud, Atef Hamada

**Affiliations:** 1 Mechanical Engineering Department, Faculty of Engineering at Shoubra, Benha University, Cairo 11629, Egypt; 2 Kerttu Saalasti Institute, Future Manufacturing Technologies (FMT), University of Oulu, FI-85500 Nivala, Finland; 3 Steel Technology Department, Central Metallurgical Research and Development Institute (CMRDI), Helwan 11722, Egypt; 4 Materials and Mechanical Engineering, Centre for Advanced Steel Research, University of Oulu, FI-90014 Oulu, Finland; 5 Department of Welding Technology and Inspection, Central Metallurgical Research and Development Institute, El-Tebbin, Helwan 11421, Egypt

**Keywords:** medium-Mn stainless steel, NiCr stainless steel, low-carbon steel, fusion welding, mechanical performance

## Abstract

In this work, 2 mm thick medium-Mn austenitic stainless steel (MMn–SS) plates were joined with austenitic NiCr stainless steel (NiCr–SS) and low-carbon steel (LCS) using the gas tungsten arc welding technique. A precise adjustment of the welding process parameters was conducted to achieve high-quality dissimilar joints of MMn–SS with NiCr–SS and LCS. The microstructural evolution was studied using laser scanning confocal and electron microscopes. Secondary electron imaging and electron backscatter diffraction (EBSD) techniques were intensively employed to analyze the fine features of the weld structures. The mechanical properties of the joints were evaluated by uniaxial tensile tests and micro-indentation hardness (H_IT_). The microstructure of the fusion zone (FZ) in the MMn–SS joints exhibited an austenitic matrix with a small fraction of δ-ferrite, ~6%. The tensile strength (TS) of the MMn–SS/NiCr–SS joint is significantly higher than that of the MMn–SS/LCS joint. For instance, the TSs of MMn–SS joints with NiCr–SS and LCS are 610 and 340 MPa, respectively. The tensile properties of MMn–SS/LCS joints are similar to those of BM LCS, since the deformation behavior and shape of the tensile flow curve for that joint are comparable with the flow curve of LCS. The H_IT_ measurements show that the MMn–SS/NiCr–SS joint is significantly stronger than the MMn–SS/LCS joint since the H_IT_ values are 2.18 and 1.85 GPa, respectively.

## 1. Introduction

Medium-Mn stainless steel (MMn–SS) is a promising material for various engineering structural applications, promoting ultra-high strength and moderate ductility [1,2]. Metallurgically, these types of steels, represented in the series 200 stainless steel category, have a metastable austenitic structure, i.e., low stacking fault energy [3,4,5]. Economically, due to the increasing price of nickel, MMn–SS has fascinated researchers in the last decade and is being utilized as a cheaper alternative in the replacement of austenitic NiCr stainless steels (NiCr–SS), especially when serving in moderately corrosive environments [6,7,8].

From the available literature, MMn–SS bearing N shows better mechanical properties than that made of NiCr–SS [3,9]. For instance, Hamada et al. [5] studied the correlation between the mechanical properties of MMn–SS, Type 201, and the grain size evolution after employing different reversion annealing treatments. Hence, the high demand for low-cost grades made of MMn–SS instead of NiCr–SS represents an urgent necessity in many engineering applications, especially in the automotive industry, and in steel structural fabrication [10,11]. MMn–SS micro-alloyed with V~1 wt.% and N~0.3 wt.% showed an enhanced mechanical strength, corrosion resistance, and hydrogen embrittlement resistance compared to traditional NiCr–SS and twinning-induced plasticity (TWIP) steel grades [12,13]. This explains the widespread concern with the development of MMn–SS series. However, there is a lack of research considering the mechanical behavior of welded joints made of MMn–SS and NiCr–SS.

Low-C steel (LCS) is widely applied in several structural engineering applications, such as the automotive industry, bridges, and other structural fields, due to its moderate comprehensive mechanical properties, reliable cost-effectiveness, and weldability [14]. The joint manufacturing between MMn–SS, a high-alloying steel grade, and LCS, a low-alloying steel grade, has to be investigated and addressed in particular, so as to increase the longevity and reduce the production costs of large-scale projects.

Fusion welding is the most versatile joining process in stainless steel assembly [15]. However, fusion welding always strongly impacts the microstructure and mechanical performance of the welded joints, in terms of solidification rates and the accompanying grain size after the welding [16,17]. Considerable efforts related to the welding of MMn–SSs have been made to investigate the effects of welding parameter changes in the metallurgical characteristics and mechanical properties of the welded joints. The effect of heat input manipulation on the characteristics of similar and dissimilar MMn–SS welded joints was reported in the literature [11,17,18,19,20,21]. It was found that with the increase in the heat input, the mechanical performance of the welded joints deteriorated. Hamada et al. [10] studied the mechanical properties and stretch-formability properties of butt joints between MMn–SS and high-strength LCS, welded using lasers at different energy inputs. They found that the mechanical strength of the processed joints is related to the softer metal, i.e., low-alloy C-steel. In the following study by Hamada et al. [22], the effect of post-weld heat treatment (PWHT) at 700 °C on the microstructure and mechanical properties of the same dissimilar joints of MMn–SS and high-strength LCS was studied. The PWHT induced strengthening effects in the BMs, as well as the welded joints.

The fusion zone (FZ) of welded joints comprising austenitic stainless steel filler metals showed austenite matrix and δ-ferrite structures [8]. In addition, it was reported that the presence of δ-ferrite ~ (3–10 vol.%) in the austenitic matrix of the FZ can preclude solidification cracks, as well as modifying the mechanical performance and corrosion resistance of welded joints [8,23,24,25]. Therefore, it has been recommended to perform comprehensive studies on the FZ produced by SS filler metals in order to understand the metallurgical nature of the δ inside the austenitic matrix [26,27].

Steel of the 201 brand is a low-Ni, medium-Mn austenitic stainless steel, and it is considered a promising material for different structural applications. The purpose of the present study, from the perspective of structural applications, is to perform a metallurgical characterization of butt joints produced by the dissimilar welding of MMn–SS with NiCr–SS and LCS by the gas tungsten arc welding (GATW) technique. It is well established that dissimilar joins are mainly employed in modern power plants, as reported by Bhanu et al. [28]. The mechanical properties of the different joints have been explored to reveal their mechanical strength. The microstructural evolution analysis was followed by comprehensive observations using the EBSD technique to identify the phase constituents. Moreover, this study compared the micro-indentation hardness of different zones in the weldments. The relationship between the mechanical performance change and the behavior of the phase structure during welding has been established, which is of great relevance to low-Ni, medium-Mn austenitic stainless steel welding.

In the present work, MMn–SS (type ASTM 201 stainless steel) is an austenitic Cr–Mn, low-Ni stainless steel, in which Ni reduction is substituted with Mn addition. Hence, for economic reasons, this steel replaces NiCr–SS in several industrial applications in emerging markets. Moreover, the mechanical properties of the MMn–SS are significantly better than those of the NiCr–SS, as shown in the above survey. This encourages steel manufacturers to replace NiCr–SS with MMn–SS. Therefore, the dissimilar welding of MMn–SS with the most commonly used steels (NiCr–SS and LCS) is necessary for promoting cost-effective, lighter, and more efficient structural joints.

It is well known that in a modern car body, dissimilar joints of different steel classes with high-strength steel and low-strength steel, e.g., low-alloyed (C-steel) and high-alloyed steels, are processed by welding. Crashworthiness necessitates high-strength and high-ductility materials in crash-relevant areas. Hence, multi-material designs are achieved in the automotive industry by combining different steels, such as austenitic steel and carbon steel. MMn–SS is becoming increasingly attractive in the automotive industry sector compared to austenitic stainless steel grade AISI 304 due to its superior strength–ductility balance and cost-effectiveness. Hence, the dissimilar welding joining of MMn–SS with conventional austenitic stainless steel (304) and LCS is gaining attention. However, the high Mn content in steel and fusion-welded joints represents a challenge, since it leads to unstable mechanical behavior in the joint. Within this context, this study makes a further contribution to the understanding of the microstructural evolution and mechanical performance of the joints of MMn–SS with NiCr–SS and LCS.

Hence, the present study aims to address the microstructural evolution and mechanical strength of various MMn–SS joints with NiCr–SS and LCS, so as to show the feasibility of fabricating high-strength joints of MMn–SS at a low cost. The microstructural analysis of the weldments was followed by comprehensive observations using different techniques: SE imaging and EBSD. The tensile properties and hardness measurements of the various MMn–SS weldments have been explored to reveal their mechanical strength.

## 2. Experimental Procedure

### 2.1. Base Metals (BMs)

The experimental BMs were commercial sheets of steel: MMn–SS, NiCr–SS, and LCS. The steels were received in the form of sheets with a thickness of 2 mm. The sheets were cut into rectangles with dimensions of 100 × 300 mm^2^. The chemical compositions of the BMs were measured by optical emission spectroscopy (model: FOUNDRY-MASTER Pro, OXFORD INSTRUMENTS, High Wycombe, UK). Table 1 illustrates the chemical compositions of the BMs and the filler metals that were utilized during the processing of the various weldments.

### 2.2. Welding Experiment

The metal joining process was conducted by employing the gas tungsten arc welding (GTAW) technique via the ESAB Tig 4300i AC/DC. During welding, the BMs were fixed by tack welds at their corners. Table 2 illustrates welding parameters such as the applied current and voltage. A pure argon shielding gas was used (purity 99.8%) with a flow rate of 15 L/min to protect the weldments from oxidation. The heat input was calculated according to Equation (1) [21]:

(1) $$\mathrm{Heat}\;\mathrm{input}\;(\mathrm{kJ}/\mathrm{mm})=\frac{\eta \;\times \;I\;\times E}{S\;\times \;1000}$$

where η represents the arc efficiency of the GTAW process, taken as 0.6 according to BS EN 1011-1 [29]. The symbols (I), (E), and (S) represent the welding current in amperes (A), arc voltage in volt (V), and welding speed in mm/s. Table 2 lists the calculated heat input of the GTAW technique according to the applied welding parameters. The quality of the weld plate was checked via a non-destructive test (radiographic inspection) and reported in our previous article; see Ref. [21]. In general, the quality of the weld plate was relatively good and without solidification cracks.

Two different filler metals, ER308L and ER309MoL, were used to form the dissimilar weldments of MMn–SS/NiCr–SS and MMn–SS/LCS, respectively. The tensile strength of both fillers was 610 MPa. It is worth mentioning that the filler ER309MoL bearing a high content of Mo, 2.5 wt.%, was used in the welding joints of LCS to induce a certain fraction of ferrite (3–10%) in the microstructure of the weldment.

### 2.3. Microstructure Characterization

The microstructures of the FZ and heat-affected zone (HAZ) were studied using secondary electron imaging (SE) and electron backscattering diffraction (EBSD) techniques, using field-emission gun scanning electron microscopy (FEG-SEM) (model: Carl Zeiss Ultra plus: Oberkochen, Germany). The EBSD maps were obtained at an accelerating voltage of 15 kV and a working distance of 12–15 mm. The butt joint weldments were sectioned perpendicularly to the welding direction. Typical metallography preparation steps were carried out, starting with mechanical grinding using SiC papers; then, the ground surfaces were polished using a diamond paste suspension and, finally, the specimens were chemically polished using a colloidal suspension of silica. The Thermo-Calc software program (Solna, Sweden) version 2022 with the TCFE9 database was employed to predict the equilibrium phases in the FZ after the welding process.

### 2.4. Mechanical Properties

A micro-indentation tester (CSM instruments: Needham, MA, USA) equipped with a diamond Berkovich indenter was used to measure the indentation hardness (H_IT_) of the different zones in the weldment, e.g., FZ, HAZ, and BM. The hardness tests were conducted under an accelerated load from zero to the maximum force (2 N) with a holding time of 10 s; in this stage, the loading behavior was recorded. Subsequently, the applied load was removed with the unloading rate of 66.6 mN/s to record the unloading behavior. The Oliver–Pharr method was automatically applied to analyze the slopes of the loading–unloading curves at the maximum force (2 N) to obtain the H_IT_ values of the weld zones [30].

The mechanical tensile properties of the BMs and the welded joints were evaluated using a hydraulic universal testing machine (UH-F1000kNI SHIMADZU: Kyoto, Japan) at a quasi-static strain rate of 10^−3^ s^−1^ at room temperature. Dog bone-shaped specimens were prepared with a gauge length of 50 mm according to the ASTM standard E8 [31]. Three tensile samples for each dissimilar joint were evaluated. The average tensile results have been provided.

## 3. Results

### 3.1. Microstructure of the BMs

**Figure 1:**
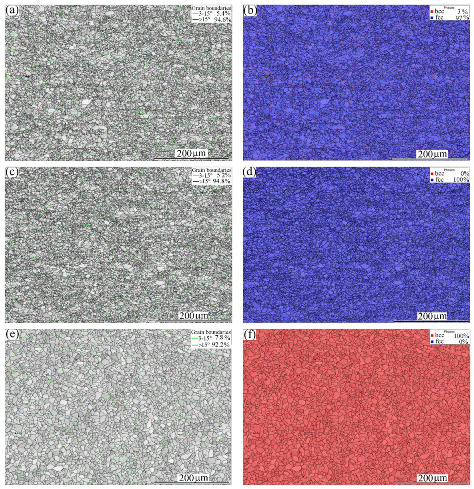
EBSD maps of the microstructures of the BMs: (**a**) IQ map of MMn–SS, (**b**) corresponding phase map of (**a**), (**c**) IQ map of NiCr–SS, (**d**) corresponding phase map of (**c**), and (**e**) IQ map of LCS, (**f**) corresponding phase map of (**e**). γ—fcc austenite is denoted by blue, and α—bcc ferrite is denoted by red (colored figure).

Figure 1 shows the microstructures of the experimental materials: BMs of MMn–SS, NiCr–SS, and LCS. Both MMn–SS and NiCr–SS exhibited similar image quality (IQ) maps and phase maps with a blue color, i.e., a fully austenitic γ-fcc structure. The MMn–SS exhibited a fine grain structure with an average grain size of 8.5 ± 4 μm, as shown in Figure 1a. About ~5% of low-angle grain boundaries (LAGBs < 15°) were promoted in the structure. The corresponding phase map shown in Figure 1b displays a small fraction of α′-martensite, ~3%, that coexists with the austenitic matrix. This martensite formation in the initial structure of MMn–SS is attributable to the mechanical polishing, which promoted strain, and consequently martensite was induced, as reported in the literature [32,33].

The NiCr–SS exhibited a fully austenitic structure with a grain size of 6 ± 2 µm, which is relatively finer than that of the MMn–SS. A small fraction of LAGBs (~5%) appeared in the grain structure, as displayed in Figure 1c. The corresponding phase map is shown in Figure 1d. The γ-fcc structure was only indexed by EBSD in a blue color.

The LCS showed a different phase structure compared with MMn–SS and NiCr–SS, as shown in Figure 1e,f. A ferritic matrix with a grain size of 10 µm is displayed. The LCS contained a small fraction of pearlite, about 7%. However, the pearlite phase was not indexed by EBSD due to the low sensitivity of the EBSD towards the pearlite structure [34]. Hence, the phase map of the LCS exhibits one phase (ferrite), which is represented in red in Figure 1f. A higher fraction of LAGBs, ~8%, was present in the grain structure of the LCS, as shown in Figure 1e.

### 3.2. Microstructural Characteristics of the Welded Joints

#### 3.2.1. Dissimilar Welded Joint of MMn–SS and NiCr–SS (MMn–SS/NiCr–SS)

**Figure 2:**
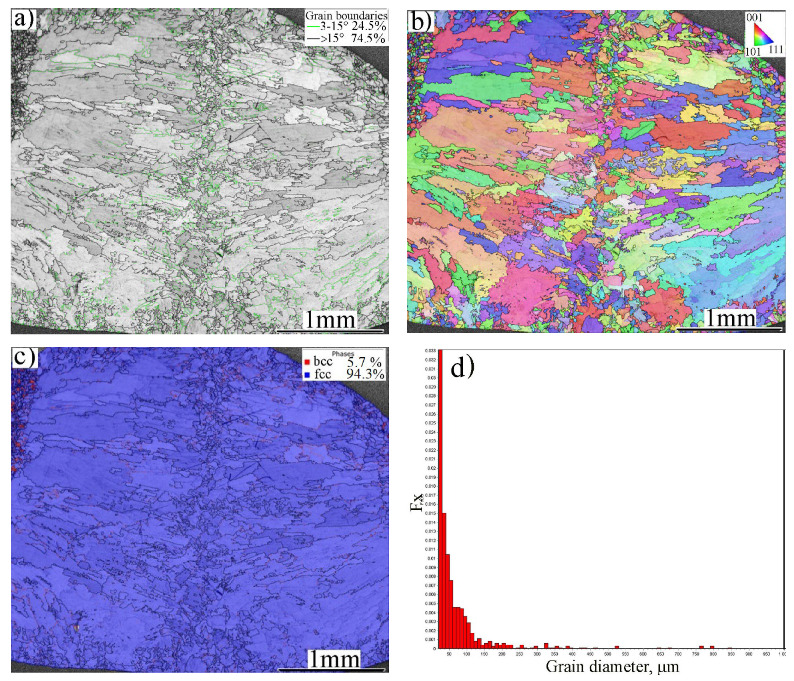
A general view of the grain structure of the FZ of the dissimilar butt joint of MMn–SS and NiCr–SS: (**a**) EBSD—IQ map, (**b**) EBSD—IPF map, (**c**) phase map, and (**d**) grain size distribution of the gains in (**a**).

Figure 2 shows a general view of the microstructure of the FZ in the dissimilar joint of MMn–SS and NiCr–SS at a low magnification, taken via EBSD. The FZ comprised coarse columnar grains and fine equiaxed grains, owing to the heterogeneous thermal distribution during the welding process. The FZ structure mainly comprised coarse columnar grains of size 500 μm near to the BM, and equiaxed grains with an average grain size of ~40 µm in the middle of the FZ, ahead of the columnar front, as shown in Figure 2a. This morphology of the weld structure can be attributed to the directional solidification from the edges of the weld pool towards the center [35]. A striking feature of the grain boundaries in the weld structure is the high presence of LAGBs, ~25%, as indicated by the green boundaries in Figure 2a.

Figure 2b displays an inverse pole figure (IPF) of the structure in the FZ, which indicates a random crystallographic orientation of the grains. Figure 2c shows that the phase structure of the FZ was mainly austenite (γ-fcc) with a small fraction of ferrite (δ-bcc), while the volume fraction of the δ-ferrite was approximately ~5.7%, as measured by EBSD. The grain size distribution of the grain structure of the FZ is shown in Figure 2d. It can be seen that 90% of the grains had sizes smaller than 100 μm. Few grains had coarse sizes of 200–800 μm.

**Figure 3:**
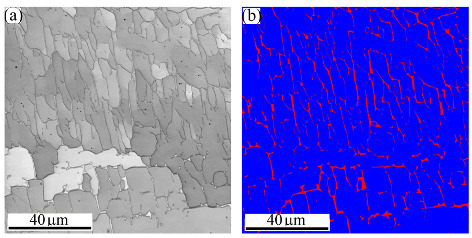
A magnified view of the microstructure of the FZ in the dissimilar butt joint of MMn–SS and NiCr–SS: (**a**) EBSD—IQ map; (**b**) EBSD-phase map (γ—fcc in blue; α—bcc in red).

Figure 3 shows a magnified view of the FZ microstructure at the middle zone of the grain structure. Figure 3a displays a dual-phase structure: γ-austenite + δ-ferrite. Sabzi et al. [36] reported a similar dual-phase structure of the weld zone in the joint of two alloys of stainless steel (316L/310S). The IQ map shows various grey levels for the austenitic grains owing to the different crystallographic textures of the grains. The regularity of the δ-ferrite distribution is a conspicuous feature of the weld structure, since it was promoted with two morphologies: ferrite laths at austenite grain boundaries and vermicular ferrite within the austenitic grains, as shown in the phase map, Figure 3b. A detailed investigation of the morphologies of the austenite and ferrite, undertaken via SEM, is presented in Section 4.

**Figure 4:**
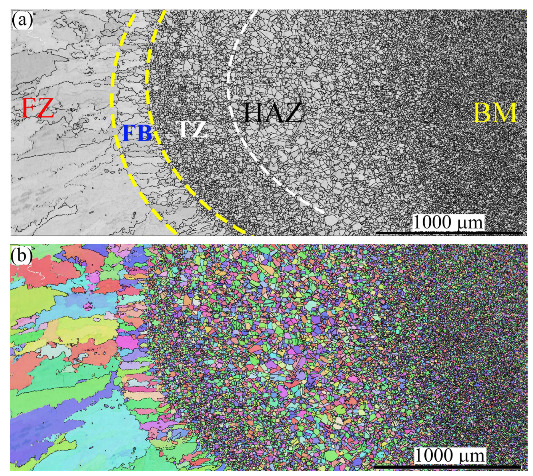
The microstructure of the HAZ, TZ, and FB near the BM NiCr–SS side: (**a**) IQ map; (**b**) IPF map.

Figure 4 mainly shows the grain structures of three zones related to the BM NiCr–SS, e.g., the HAZ, transition zone (TZ), and fusion boundary (FB), which is a partially melted zone (PMZ) at the interface between the FZ and the BM, highlighted by the yellow dashed lines. Homogeneity in the sizes and grains of the FB along the weld can be observed. Another zone in the fine grain structure, the TZ, highlighted by the white dashed line, appeared between the FB and the HAZ. The mechanism promoting TZs in the stainless-steel BMs is discussed in Section 4.

The width of the FB was relatively small compared to the other zones. For instance, the sizes of the HAZ, TZ, and FB were ~1400, 350, and 225 μm, respectively. No phase transformation occurred since the austenite was the dominant structure in the FB, TZ, and HAZ. It is noteworthy that the annealing twins disappeared in the FB, while they remained noticeable in the TZ and HAZ. The twin plates were reoriented in a similar manner as the parent matrix when the temperature reached 1000 °C (known as the de-twinning phenomenon) [37,38]. Therefore, the disappearance of the twin plates in the FB confirms that the temperature there exceeded 1000 °C, while the existence of the twin plates in the TZ confirms that the temperature there was less than 1000 °C.

The grain structure of the FB consisted of columnar grains oriented towards the BM. This is attributed to the unidirectional heat flux from the molten pool in the FZ towards the BM, as reported in the literature [39]. However, fine equiaxed grains can be observed in the TZ. The transition of the columnar structure in the FB into fine equiaxed grains in the TZ is discussed in Section 4. As expected, a coarse grain structure was promoted in the HAZ, as shown in Figure 4a. The corresponding IPF map shown in Figure 4b indicates that the grains in these zones preferred <101> and <111> crystallographic orientations.

#### 3.2.2. Dissimilar Welded Joint of MMn–SS and LCS (MMn–SS/LCS)

**Figure 5:**
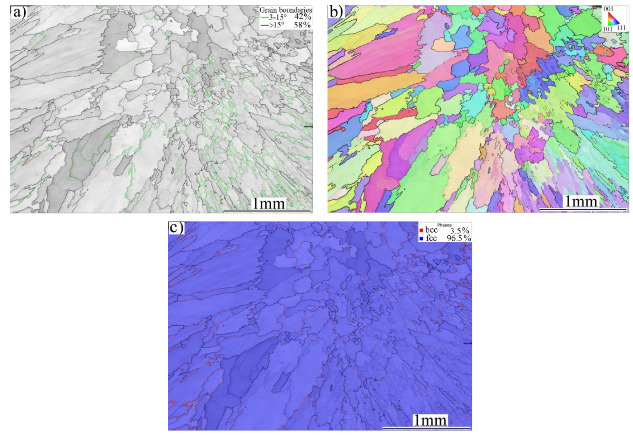
Microstructure of the FZ of the dissimilar butt joint of MMn–SS and LCS: (**a**) IQ—map, (**b**) IPF map, and (**c**) phase map (γ—fcc in blue; δ—bcc in red).

Figure 5 exhibits a general view of the microstructure of the FZ of MMn–SS/LCS. Figure 5a shows the IQ map of the grain structure, which displays irregular and columnar grains. Meanwhile, Figure 5a shows that a significant fraction of LAGBs, ~42%, was induced in the weld structure. This is presumably attributable to the residual stresses promoted in the welding during fast cooling [40,41]. The corresponding IPF map shown in Figure 5b indicates that the orientation of the grains in the FZ preferred <101> and <001> crystallographic orientations. The phase map in Figure 5c shows a dual-phase structure, since the structure is mainly austenite with a few fractions of δ-ferrite, about 3.5%, as calculated by EBSD.

**Figure 6:**
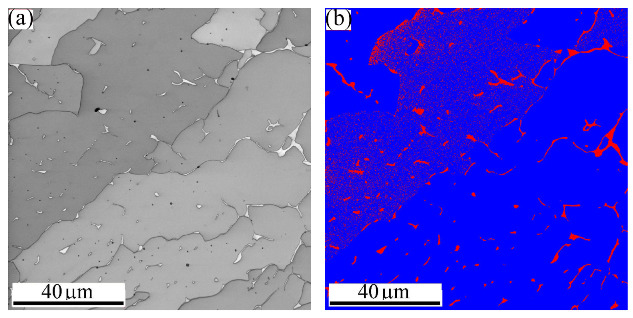
High magnification of the microstructure of the FZ in the dissimilar butt joint of MMn–SS and LCS via EBSD: (**a**) IQ map; (**b**) phase map (γ—fcc in blue; α—bcc in red).

Figure 6 displays a magnified view of the microstructure of the FZ of the MMn–SS/LCS joint. Large dendritic columnar austenitic grains of dark grey colors are shown in the IQ map in Figure 6a. White thin precipitates of the δ-bcc phase with an acicular morphology were mainly located in the inter-dendritic regions of the austenite phase. This is confirmed by the corresponding phase map in Figure 6b, which depicts the matrix austenitic grains in blue and the δ-ferrite in red. It can be observed that δ-ferrite with different morphologies is promoted inside the austenitic grains, as highlighted by the yellow dashed ovals. Similarly, Chuaiphan et al. [42] reported a duplex structure of austenite and δ-ferrite in the microstructures of the FZ in the joint of 304-SS and LCS grade 1020 welded via the GTAW technique.

**Figure 7:**
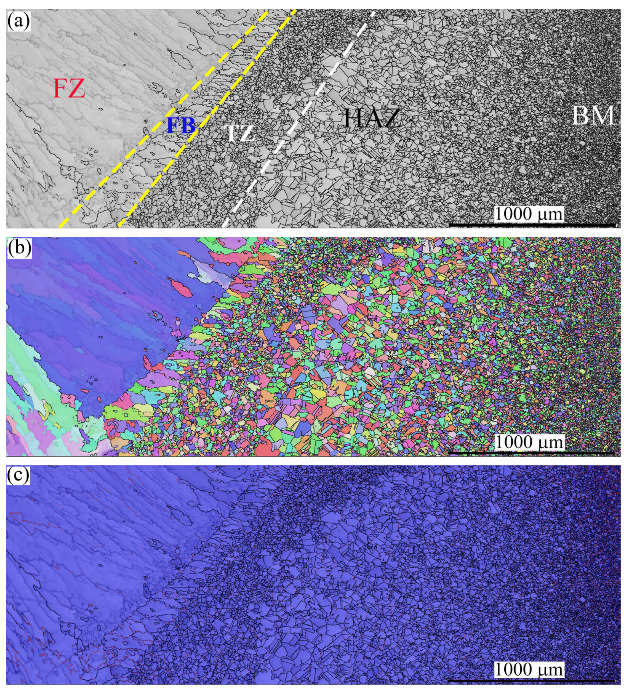
The microstructure of the FZ, PMZ, TZ, HAZ, and BM in the MMn–SS side after the dissimilar welding of MMn–SS and LCS: (**a**) IQ map, (**b**) IPF map, (**c**) phase map (austenite γ—*fcc* in blue).

Figure 7 shows the microstructures of the FZ, PMZ (FB), TZ, HAZ, and BM on the MMn–SS side via EBSD. Figure 7a shows the IQ map of the grain structure. Irregular and columnar grains are shown. Meanwhile, Figure 7b displays the corresponding IPF map of the orientation of the grains in the FZ and HAZ, exhibiting different crystallographic orientations. Figure 7c displays the corresponding phase map, wherein the structure of the HAZ is shown to have been mainly austenite.

The microstructure of the MMn–SS displays an FB with a width of 0.216 ± 0.06 mm. Moreover, the TZ showed a width of 0.424 ± 0.1 mm, while the HAZ showed a width of 1.8 ± 0.3 mm. The width of the coarse grain HAZ (CGHAZ) was 1.1 ± 0.1 mm, with an average grain size of 60 µm, while the width of the fine grain HAZ (FGHAZ) was 0.7 ± 0.1 mm, with an average grain size of 25 µm.

**Figure 8:**
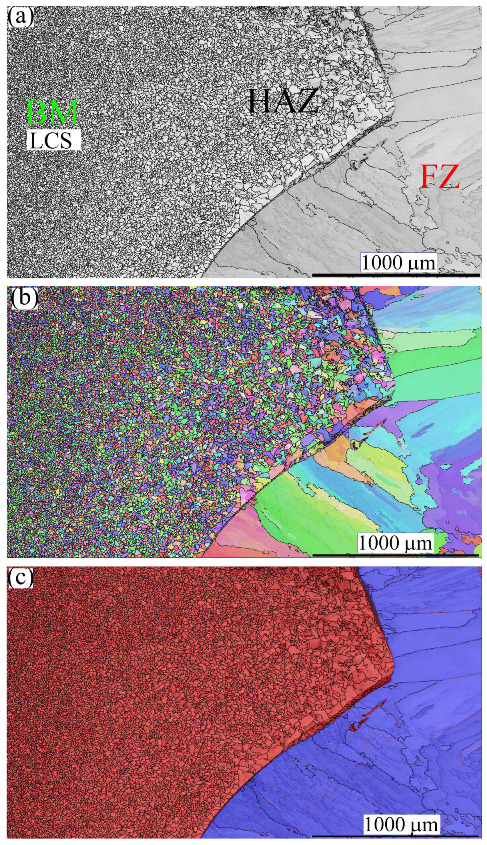
The microstructures of the FZ, PMZ, HAZ, and BM in the LCS side after dissimilar welding of MMn–SS and LCS: (**a**) IQ map; (**b**) IPF map; (**c**) phase map (ferrite in α—bcc in red and austenite γ—*fcc* in blue).

Figure 8 shows the microstructures of FZ, PMZ, HAZ, and BM on the LCS side, taken via EBSD. Figure 8a displays an IQ map of the grain structure. The columnar grain structure was limited at the CGHAZ on the LCS side, while an equiaxed structure was promoted. Figure 8b exhibits the corresponding IPF map of the orientation of the grains in the FZ and HAZ, with different crystallographic orientations. Figure 8c shows the corresponding phase map, wherein it is clear that the structure in the HAZ is mainly α-ferrite (colored in red).

Furthermore, the LCS did not show a TZ such as that found after the welding of the stainless steel joints. Moreover, the microstructure of the LCS showed a very thin PMZ with a width of 0.016 ± 0.008 mm. The HAZ showed a width of 2 ± 0.15 mm. The average grain size in CGHAZ was measured at 55 ± 25 µm, while the average grain size in the FGHAZ was measured at 20 ± 10 µm.

### 3.3. Mechanical Properties of MMn–SS Weld Joints

**Figure 9:**
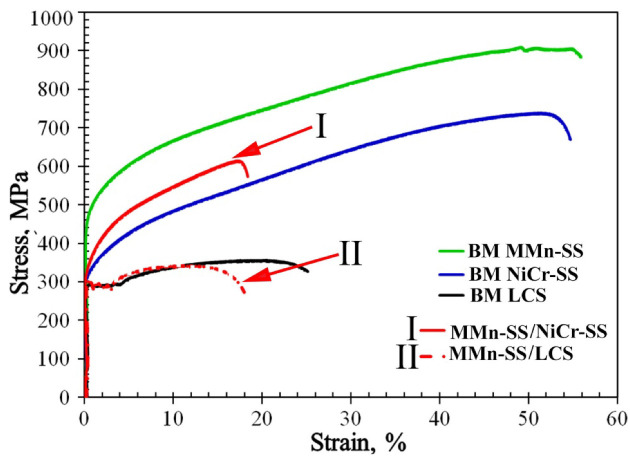
Tensile flow curves of the dissimilar MMn–SS butt joints with NiCr–SS and LCS.

The engineering tensile stress–strain curves of the MMn–SS joints with NiCr–SS and LCS at room temperature are shown in Figure 9. A striking feature of the tensile flow behavior is the considerable deformation capacity and strain hardening of the MMn–SS/NiCr–SS joint. During tensile testing, the joint was broken within the weld. Hence, the yield strength and UTS of the joint, 340 and 610 MPa, were relatively lower than those of the BMs (MMn–SS and NiCr–SS). However, the tensile flow behavior of the joint MMn–SS/LCS resembles the tensile flow behavior of the LCS: a Lüders plateau with a low yield strength. This is because the joint was broken within the LCS BM.

The mechanical tensile properties of the MMn–SS joints compared with the BMs are illustrated in Table 3. It can be observed that the mechanical strengths of the BMs, MMn–SS, and NiCr–SS were higher than those of the weldment; for instance, the UTSs of the BMs and weld were 915, 738, and 610 MPa, respectively. However, the mechanical strength of the other joint with LCS was slightly lower than that of the LCS BM, 340 and 360 MPa, respectively.

It is well known that the weld efficiency is related to the tensile strength of the processed joint. Equation (2) shows the terms for calculating the weld efficiency (WE) in both joints [43]:

(2) $$\mathrm{WE}\;(\%)=\frac{TS_{WM}}{\;TS_{BM}}\;\times 100$$

where *TS_WM_* is the UTS of the weld, and *TS_BM_* is the UTS of the weaker BM in the joint. The WE parameter of the welded joints was calculated based on the tensile properties extracted from the tensile flow curves (Figure 9). The WEs of the MMn–SS/NiCr–SS and MMn–SS/LCS joints were 82 and 95%, respectively; while the MMn–SS/NiCr–SS joint was slightly softer than the NiCr–SS BM, the MMn–SS/LCS joint exhibited a similar strength to the LCS-BM.

**Figure 10:**
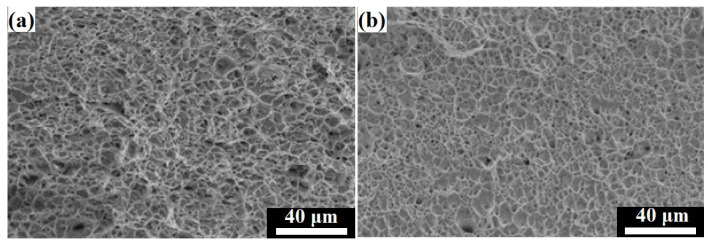
SEM fractography of the tensile specimen welded joints: (**a**) MMn–SS/NiCr–SS joint, (**b**) MMn–SS/LCS joint.

Figure 10 shows the fracture surfaces of the welded joints after tensile testing. Figure 10a,b show the fractography of the tensile specimens, MMn–SS/NiCr–SS and MMn–SS/LCS, respectively. It can be observed that the fracture surfaces of the welded joints exhibited a dimple feature with varying shapes and sizes, which is a typical characteristic of a ductile fracture.

## 4. Discussion

In the present study, the dissimilar joints of MMn–SS/NiCr–SS and MMn–SS/LCS were processed by the GTAW technique via ER308L and ER309MoL filler metals, at heat inputs of 0.486 and 0.604 kJ/mm, respectively. In this section, we explain variations in the micro-indentation hardness values in view of the microstructural evolution in the FZ and TZ of the welded joints.

### 4.1. Thermo-Calc Analysis of the Predicted Phases in the FZ

**Figure 11:**
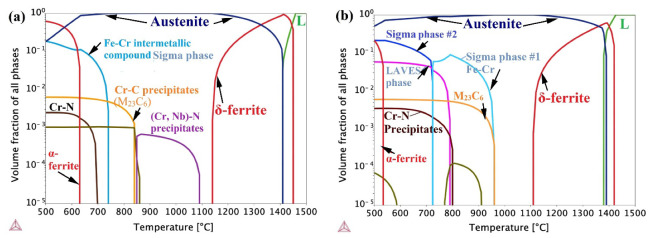
Thermo-Calc results showing equilibrium phases predicted in the FZ of (**a**) MMn–SS/NiCr–SS joint; (**b**) MMn–SS/LCS joint.

Figure 11 shows the Isopleth equilibrium phase diagrams for the chemical composition of the FZs, analyzed by EDS at several spots in the studied joints using the TCFE9 Thermo-Calc software. The volume fractions of the equilibrium phases are displayed in the temperature range of 1500 to 500 °C. It can be observed that the δ-ferrite (red line) started to form from the molten pool at 1450 and 1410 °C in the joints of MMn–SS/NiCr–SS (Figure 11a) and MMn–SS/LCS (Figure 11b), respectively. The proportions of δ-ferrite were ~97% and ~80%, mixed with 3% and 20% liquid, in the FZ of the MMn–SS/NiCr–SS and MMn–SS/LCS joints, respectively. This implies the possibility of δ-ferrite forming at a higher fraction in the MMn–SS/NiCr–SS than in the MMn–SS/LCS joint, which depends on the cooling rate after weld processing (that is related to the heat input consumed during the welding process) [21].

With cooling, the δ-ferrite and the existing liquid transformed into an austenite phase (blue line) up to complete transformation at 1150 and 1120 °C in the joints MMn–SS/NiCr–SS and MMn–SS/LCS, respectively. With cooling to the lower temperature of 500 °C, α-ferrite formed at fractions of 50% and 10% in the FZs of MMn–SS/NiCr–SS and MMn–SS/LCS, respectively. However, these phases were in equilibrium. It is well established that the welding process involves a non-equilibrium state, due to the fast cooling after the welding processing. Hence, the δ-ferrite formed at high temperatures was kept in the structures of the weldments. The Thermo-Calc plots show the formation of carbide–nitride precipitates due to the existence of the alloying elements, see Section 4.2.

It is well established that different solidification modes occur after the weld processing of austenitic steels based on the ratio of Cr_eq_/Ni_eq_. For instance, the ferritic austenitic (FA) mode refers to the solidification of ferrite, followed by austenite formation, which is promoted when 1.48 < (Cr_eq_/Ni_eq_) < 1.95 [11,23].

In the present study, the Cr_eq_ and Ni_eq_ values were calculated according to Equations (3) and (4), as follows:

Cr_eq_ = Cr + Mo + 0.5 Nb + 1.5 Si (3)

Ni_eq_ = Ni + 30 (C + N) + 0.5 (Mn + Cu) (4)

The chemical compositions of the FZs were determined by EDS attached to an SEM to calculate Cr_eq_ and Ni_eq_. On the one hand, the EDS has a low sensitivity in the detection of low-atomic number elements, such as C and N, since the sensitivity of EDS analysis is approximately 0.1 wt.% for all elements [44,45]. On the other hand, the calculation of the elements present at the FZ by EDS is accepted in numerous other works [11,13,17]. The C content in the FZs was calculated by the stoichiometry method, which depends on the quantitative relationship between the C contents of the paired steel plates (MMn–SS/NiCr–SS and MMn–SS/LCS) and the filler metals (ER308L and ER309MoL), as well as the effect of the dilution percentage for each steel in the joint. This is determined according to Equation (5), as follows [11]:

C_FZ_ = (C.D) _BM1_ + (C.D) _BM2_ + (C.D) _FM_ (5)

where C_FZ_ is the C content of the FZ, (C.D) _BM1_ represents the C content and dilution percentage of BM 1, (C.D) _BM2_ is the C content and dilution percent of BM 2, and (C.D) _FM_ represents the C content and dilution percent of the filler metal utilized. Based on the δ-ferrite content measured via EBSD, dilution percentages of 15% and 70% were assumed for the paired steels and the fillers, respectively. Consequently, the C contents in the FZs were 0.032 and 0.029 wt.% for MMn–SS/NiCr-SS and MMn–SS/LCS, respectively.

Here, the ratio of Cr_eq_/Ni_eq_ was 1.8 and 1.53 for MMn–SS/NiCr–SS and MMn–SS/LCS, respectively. This indicates that the weld pool exhibited an FA solidification mode, which means that the δ-ferrite was solidified first, as it preceded the formation of the austenite phase, and this agrees with the results of the Thermo-Calc shown in Figure 11.

The δ-ferrite volume fraction existing in the FZ of the MMn–SS/LCS joint (3.5%) was lower than that existing in the FZ of the MMn–SS/Ni-Cr-SS joint, which was 5.7%. Despite this, the filler, ER308L, utilized during the welding of the MMn–SS/NiCr–SS, had a Mo content (0.2 wt.%) lower than that of the filler ER309MoL (2.5 wt.%) used in the welding of the MMn–SS/LCS joint. It is well known that Mo is a ferrite stabilizer, as reported in the literature [46,47].

It is reasonable to assume that the change in the fractions of δ-ferrite present in the FZs of the MMn–SS/NiCr–SS and MMn–SS/LCS joints can be attributed to two factors. First, the heat input in the MMn–SS/NiCr–SS joint was lower than that in the MMn–SS/LCS joint, 0.486 compared to 0.604 kJ/mm, respectively. It is established that the higher the heat input, the lower the cooling rate [21]. Therefore, the δ-ferrite (which was solidified first, according to the FA solidification mode) was isothermally transformed into austenite in the FZ of the MMn–SS/LCS joint due to the relatively slow cooling rate compared to the cooling rate of the MMn–SS/NiCr–SS. Abioye et al. [48] reported that the volume fraction of the δ-ferrite phase increases as the heat input decreases during the dissimilar welding of NiCr–SS (304) with LCS. Second, the high alloying element, Cr, in the FZ of the MMn–SS/NiCr–SS, promotes a higher δ-ferrite content than that in the MMn–SS/LCS joint.

### 4.2. Characteristics of δ-Ferrite in the FZs

**Figure 12:**
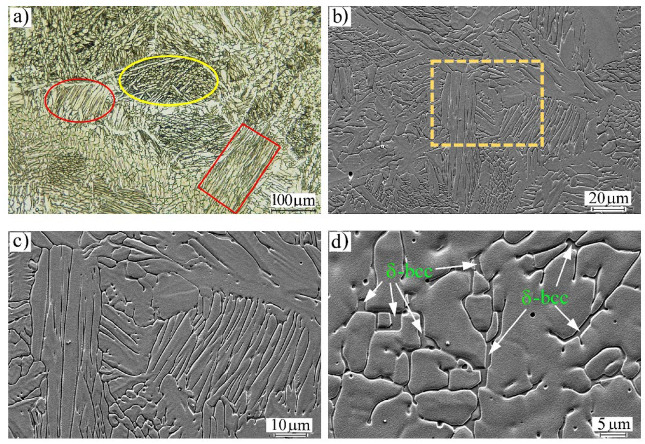
Microstructures of the FZ in the dissimilar welded joint MMn–SS/NiCr–SS: (**a**) general view taken by laser focal imaging, (**b**) SEM imaging, (**c**) a magnified view of the dashed box in (**b**) showing the different morphologies of the austenite grains, and (**d**) a magnified view of the dendrite δ-ferrite.

Figure 12 shows the dendritic structure of the FZ, determined by laser microscopy and SEM imaging. A conspicuous feature of the grain structure in the FZ can be observed, i.e., the irregularity of the morphology of the grains, as shown in Figure 12a. For instance, the columnar austenitic dendritic morphology, highlighted in red, and the equiaxed cellular grains, highlighted in the yellow oval, are promoted in the FZ. It can be confirmed that the FZ structure was composed of γ-austenite and δ-ferrite phases, as seen in magnified views taken by SEM; Figure 12b–d. Figure 12c reveals a “Widmanstätten pattern” in the present phases, i.e., lamellar austenite grains and parallel acicular ferrite located at the austenite grain boundaries. Ferrite was present with two morphologies, ferrite laths at the austenite grain boundaries and vermicular ferrite within the austenitic grains, similar to what has been reported in the literature [6]. Figure 12d shows a higher magnification of the FZ structure, exhibiting that other parts of the δ-ferrite were located inside the bulk of the austenite grains and had branched formations. The Widmanstätten ferrite lamellas were very thin, <1 μm (which agrees with the results presented by EBSD in Figure 3b).

**Figure 13:**
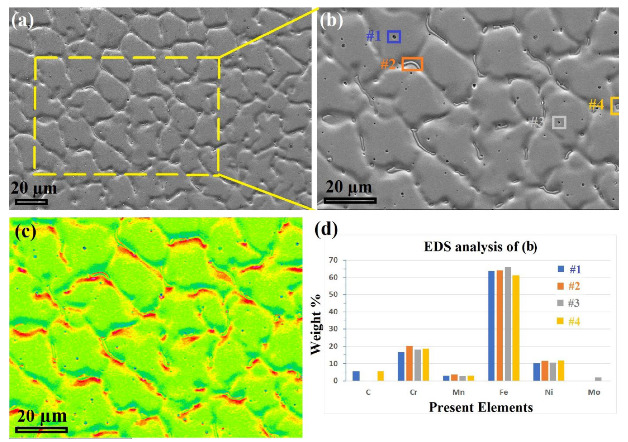
Detailed views of the MMn–SS/LCS weld via SEM, showing (**a**) the FZ, (**b**) magnified section of the dashed yellow rectangle in (**a**), (**c**) elemental distribution map of (**b**), and (**d**) EDS analysis of the four colored boxes in (**b**).

Figure 13 shows magnified views of the FZ in the MMn–SS/LCS welded joint, a map analysis of the present elements, and the corresponding EDS analysis performed via SEM. Figure 13a,b display intermittent δ-ferrite at the grain boundaries of the austenite, as well as precipitates of carbides, which appeared randomly at the grain boundaries and inside the grains of the austenite phase. Figure 13c exhibits the elemental distribution map of the elements present in the microstructure; the map shows darker colors in intermittent forms on the grain boundaries at the locations of the δ-ferrite. The presence of the δ-ferrite phase with an intermittent shape confirms that the transformation of the δ-ferrite into austenite was enhanced due to the slow cooling rate associated with the relatively high heat input applied during the welding processing. This agrees with the findings in the literature [11], which reported that with the rise in the cooling rate, lower percentages of δ-ferrite are transformed into austenite. This result highlights the serious effects of the heat input entered during the welding of MMn–SS joints, since it directly affects the amount of δ-ferrite present in the FZ.

The EDS analysis (Figure 13d) shows that the δ-ferrite contained high amounts of Cr (~20 wt.%). On the other hand, the precipitated carbides on the grain boundaries contained C and Cr, while the carbides precipitated inside the austenite matrix contained C, Cr, and Mo. The EDS analysis of spot #3, shown in Figure 13d, displays that the micro-scale pitting/porosity on the surface was promoted in the FZ, which was related to the high heat input employed during the welding, and this is consistent with the literature [49].

### 4.3. Appearance of the TZ

**Figure 14:**
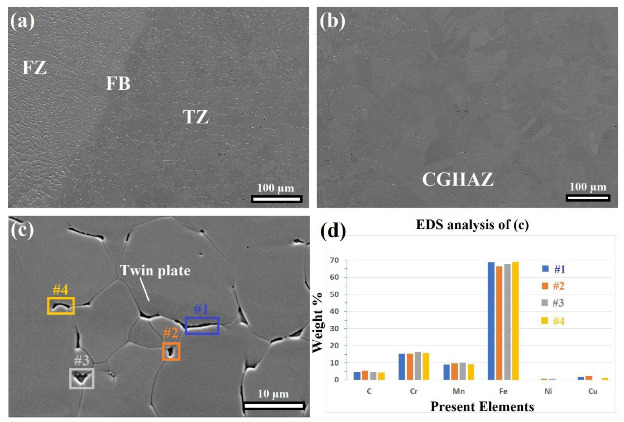
SEM images of the microstructural evolution on the LCS side showing: (**a**) the FZ, FB, and TZ; (**b**) CGHAZ; (**c**) the magnified section of the TZ, and (**d**) the EDS analysis of the four spot-points in (**c**).

Figure 4 and Figure 7 show the appearance of the TZ between the FBs and HAZs of the NiCr–SS and MMn–SS sides, while Figure 8 does not indicate the existence of any TZ on the LCS side. As reported in the Results section, the grain size in the TZ was finer than the grain size of the neighboring CGHAZ. Therefore, a detailed microstructural investigation of the TZ in the MMn–SS side is presented in Figure 14, which explains the formation of the fine grain structure there.

Figure 14a shows a formation of longitudinal laths or fine precipitates along the grain boundaries of the austenite matrix in the TZ. Figure 14b confirms that those precipitates did not appear at the CGHAZ, while a grain coarsening effect was promoted. Figure 14c displays a magnified view of the TZ; it shows that the precipitates are located at the grain boundaries of the austenite phase. Furthermore, twin plates appear inside the austenite grains; they were stable despite the heat passing from the FZ towards the BM, which confirms that the temperature in the TZ was less than 1000 °C [37,38].

The main reason behind the appearance of the TZ is the sensitization effect [50,51,52]. The sensitization takes place in the HAZ region at a temperature range of 400 to 800 °C after the welding of austenitic steels. It refers to the Cr-depletion of the zones close to the grain boundaries, which is followed by a formation of Cr-rich chains at the grain boundaries of the austenite matrix, whereas C is diffused out of the austenite grains to enhance the formation of C-Cr precipitates along the grain boundaries. However, the formation of the C-Cr-rich chains along the grain boundaries negatively affects the corrosion behavior of austenitic steels [53]. Furthermore, according to the results presented in Figure 11, chromium-carbides started to precipitate at 850 and 950 °C for MMn–SS/NiCr–SS and MMn–SS/LCS, respectively.

Figure 14d exhibits the chemical analysis of the precipitates (located at the grain boundaries) via the EDS; it confirms that they are Chromium carbides, as it mainly contains C, Cr, and Mn elements. It was reported that the precipitation of carbide particles on the grain boundaries has pinning effects on the grain growth [54,55,56]. Therefore, the precipitated carbides in the TZ work as obstacles for the movement of the grain boundaries, which hinders the coarsening of the grains size at the TZ and works as heterogeneous nucleation sites, which enhances the formation of fine equiaxed structures in the TZ; therefore, the size of the grains at the TZ was finer than the grains which exist at the FB and CGHAZ, which is similar to the results presented in the literature [52,57,58].

### 4.4. Evolution of Micro-Indentation Hardness Values

**Figure 15:**
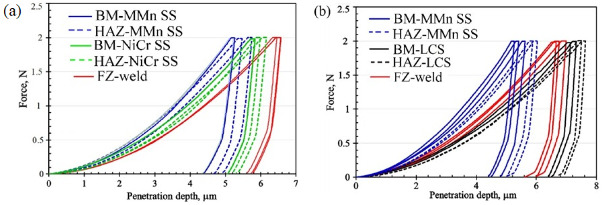
Indentation load–penetration depth curves, loading–unloading curves of different zones: (**a**) dissimilar welding of MMn/NiCr-SS, and (**b**) dissimilar welding of MMn/LCS (colored figure).

Micro-indentation hardness tests of the welded joints were carried out to evaluate the effect of the present phases in the microstructure, as well as grain size variations, on the evolution of the hardness in the welded joints. Figure 15 presents the welded joints’ typical micro-indentation load–penetration depth (P–h) curves for different zones (BM, HAZ, and FZ). The penetration load was kept constant at 2 N. The variation in penetration depths across the same weldments is attributed to the microstructural and compositional inhomogeneity.

The BM MMn–SS recorded a minimum penetration depth of 5.4 ± 0.2 µm (corresponding to H_IT_ = 3.16 ± 0.13 GPa), while the BM NiCr–SS recorded a penetration depth of 5.75 ± 0.1 µm (corresponding to H_IT_ = 2.81 ± 0.01 GPa). Despite the NiCr–SS showing a grain size (6 µm) that was finer than that of the MMn–SS (8.5 um), the MMn–SS had a higher content of interstitial atoms, such as C and N. For instance, the C and N contents in the MMn–SS BM were 0.1 and 0.2 wt.%, respectively, while the corresponding C and N contents in the BM NiCr–SS were 0.03 and 0.1 wt.%, respectively. Furthermore, the BM MMn–SS had a higher tensile strength, 915 MPa, than that of the NiCr–SS, 738 MPa. Therefore, the penetration depth in the MMn–SS was smaller than that in the NiCr–SS. On the other hand, the LCS-BM recorded a penetration depth of 7.15 ± 0.1 µm (corresponding to H_IT_ = 1.6 ± 0.05 GPa). These results confirm that the ferrite phase had the lowest resistance to indentation among the present phases.

The penetration depth in the FZ of the stainless steel weldment, MMn–SS/NiCr–SS, was measured as 6.5 ± 0.1 µm, which is greater than the penetration depths of the BMs and HAZs, and corresponds to H_IT_ = 2.18 ± 0.1 GPa. This decrease in the hardness is related to the grain coarsening effect in the FZ, since the grains’ sizes in the FZ were greater than their counterparts in the BMs and the HAZs. On the other hand, the FZ of the MMn–SS/LCS joint showed a penetration depth of 6.7 ± 0.15 µm (corresponding to H_IT_ = 1.85 ± 0.1 GPa), which was softer than the hardness of the FZ of the MMn–SS/NiCr–SS joint (2.18 ± 0.1 GPa). However, the FZ in the Mn-SS/NiCr–SS joint exhibited a higher fraction of the soft phase of δ-ferrite, which reduced the hardness, as reported in Ref. [59]. However, the higher H_IT_ of the FZ in the Mn-SS/NiCr–SS joint was mainly attributed to the solid solution strengthening mechanism, related to the high presence of alloying elements (Ni, Cr, Fe) promoted by combing three high-alloyed steels (MMn–SS, NiCr–SS, filler ER308L). This is in agreement with the findings of Karimi et al. [60]. Furthermore, the HAZ of the LCS side exhibited the highest penetration depth of 7.55 ± 0.1 µm, corresponding to H_IT_ = 1.45 ± 0.05 GPa. This can be attributed to the coarsening of the grain structure in HAZ.

## 5. Conclusions

The joints of medium-Mn stainless steel with a high tensile strength of 915 MPa, NiCr stainless steel with a strength of 738 MPa, and low C-steel with a low tensile strength of 360 MPa were manufactured using the GTAW technique to assess the durability and feasibility of using low-cost joints for structural applications. Microstructural and mechanical performance (tensile strength and micro-indentation hardness) analyses of the dissimilar metal joints were performed. The main conclusions can be summarized based on the intensive experimental observations as follows:

1. The microstructures of the FZs for both joints comprised mainly austenite matrix and a small fraction of δ-ferrite (3.5~5.7%);
2. Sensitization was promoted in the HAZ of the stainless steels; hence, Cr carbides were precipitated on the grain boundaries of the austenite matrix;
3. The tensile strength of the dissimilar MMn–SS joint with NiCr–SS (610 MPa) was significantly higher than that of its counterpart joint of MMn–SS with LCS (340 MPa), since the flow stress curve of the joint was similar to that of the BM LCS, which was the weaker metal in the joint;
4. The micro-indentation hardness values of the FZs in the MMn–SS joints with NiCr–SS and LCS were 2.18 and 1.85 GPa, respectively. This was attributed to the greater strengthening of the solid solution achieved by combing the alloying elements (Ni, Cr, and Fe).

## Figures and Tables

**Table 1:** Chemical composition (in wt.%) of the BMs and filler metals used in the present study.

Materials	Elements (Weight%)										
	C	Si	Mn	Cr	Ni	Mo	Cu	V	N	Nb	Fe
MMn–SS	0.098	0.15	9.57	14.60	1.26	0.031	1.43	0.06	0.20	0.043	Bal.
NiCr–SS	0.025	0.42	1.12	18.70	7.80	0.001	0.014	0.12	0.10	0.051	Bal.
LCS	0.053	0.04	0.37	0.025	0.03	0.005	0.09	0.001	-	0.001	Bal.
ER308L	0.02	0.40	1.90	19.80	9.80	0.20	0.15	-	-	-	Bal.
ER309MoL	0.01	0.40	1.50	22.00	14.80	2.50	0.12	-	-	-	Bal.

**Table 2:** GTAW parameters of the welded joints were investigated.

Welded Joint	Pass	Current (A)	Voltage (V)	Welding Speed (mm/s)	Heat Input per Pass (kJ/mm)	Total Heat Input (kJ/mm)
MMn–SS/NiCr–SS	Root	70	12.5	2.6	0.202	0.486
	Cup	110	12.5	2.9	0.284	
MMn–SS/LCS	Root	70	12.5	1.7	0.309	0.604
	Cup	110	12.5	2.8	0.295	

**Table 3:** Quasi-static tensile properties of the MMn–SS joints with NiCr–SS and LCS. The BMs are included for comparison.

Materials	Tensile Properties				Location of Fracture
	Yield (MPa)	Ultimate (MPa)	Elongation (%)	Joint Efficiency (%)	
MMn–SS	420 ± 13	915 ± 15	54 ± 5	-	Gauge length
NiCr–SS	320 ± 10	738 ± 17	52 ± 2	-	Gauge length
LCS	303 ± 7	360 ± 8	22 ± 3	-	Gauge length
MMn–SS/NiCr–SS	340 ± 8	610 ± 10	18 ± 2	82	Weld metal
MMn–SS/LCS	280 ± 6	340 ± 5	16 ± 1	95	LCS-BM

## Data Availability

Data will be made available on request.

## References

[1] Chang Y., Han S., Li X., Wang C., Zheng G., Dong H. (2018). Effect of shearing clearance on formability of sheared edge of the third-generation automotive medium-Mn steel with metastable austenite. J. Mater. Process. Technol..

[2] Li T., Yan S., An D., Li X., Chen J. (2022). Austenite transformation associated with δ-ferrite phase in a medium-Mn steel after cold-rolling and intercritical annealing. J. Mater. Process. Technol..

[3] Hamada A., Karjalainen L., Misra R., Talonen J. (2013). Contribution of deformation mechanisms to strength and ductility in two Cr–Mn grade austenitic stainless steels. Mater. Sci. Eng. A.

[4] Kisko A., Hamada A., Talonen J., Porter D., Karjalainen L. (2016). Effects of reversion and recrystallization on microstructure and mechanical properties of Nb-alloyed low-Ni high-Mn austenitic stainless steels. Mater. Sci. Eng. A.

[5] Hamada A., Kisko A., Sahu P., Karjalainen L. (2015). Enhancement of mechanical properties of a TRIP-aided austenitic stainless steel by controlled reversion annealing. Mater. Sci. Eng. A.

[6] Chuaiphan W., Srijaroenpramong L. (2020). Optimization of TIG welding parameter in dissimilar joints of low nickel stainless steel AISI 205 and AISI 216. J. Manuf. Process..

[7] Vashishtha H., Taiwade R.V., Khatirkar R., Ingle A., Dayal R.K. (2014). Welding Behaviour of Low Nickel Chrome-Manganese Stainless Steel. ISIJ Int..

[8] Chuaiphan W., Srijaroenpramong L. (2019). Optimization of gas tungsten arc welding parameters for the dissimilar welding between AISI 304 and AISI 201 stainless steels. Def. Technol..

[9] Liu Z., Fan C., Chen C., Ming Z., Yang C., Lin S., Wang L. (2020). Design and evaluation of nitrogen-rich welding wires for high nitrogen stainless steel. J. Mater. Process. Technol..

[10] Hamada A., Ali M., Ghosh S., Jaskari M., Keskitalo M., Järvenpää A. (2021). Mechanical performance and formability of laser-welded dissimilar butt joints between medium-Mn stainless steel and high-strength carbon steel. Mater. Sci. Eng. A.

[11] Vashishtha H., Taiwade R.V., Sharma S., Marodkar A.S. (2019). Microstructural and Mechanical Properties Evolution of Bimetallic Cr-Ni and Cr-Mn-Ni Stainless Steel Joints. Met. Microstruct. Anal..

[12] Allam T., Guo X., Lipińska-Chwałek M., Hamada A., Ahmed E., Bleck W. (2020). Impact of precipitates on the hydrogen embrittlement behavior of a V-alloyed medium-manganese austenitic stainless steel. J. Mater. Res. Technol..

[13] Hamada A., Kömi J. (2018). Effect of microstructure on mechanical properties of a novel high-Mn TWIP stainless steel bearing vanadium. Mater. Sci. Eng. A.

[14] Zhang Y.-J., Chandiran E., Dong H.-K., Kamikawa N., Miyamoto G., Furuhara T. (2021). Current Understanding of Microstructure and Properties of Micro-Alloyed Low Carbon Steels Strengthened by Interphase Precipitation of Nano-Sized Alloy Carbides: A Review. Jom.

[15] Tümer M., Schneider-Bröskamp C., Enzinger N. (2022). Fusion welding of ultra-high strength structural steels—A review. J. Manuf. Process..

[16] Mirshekari G., Tavakoli E., Atapour M., Sadeghian B. (2014). Microstructure and corrosion behavior of multipass gas tungsten arc welded 304L stainless steel. Mater. Des..

[17] Bansod A.V., Patil A.P., Moon A.P., Shukla S. (2017). Microstructural and Electrochemical Evaluation of Fusion Welded Low-Nickel and 304 SS at Different Heat Input. J. Mater. Eng. Perform..

[18] Chuaiphan W., Srijaroenpramong L. (2020). Microstructure, mechanical properties and pitting corrosion of TIG weld joints alternative low-cost austenitic stainless steel grade 216. J. Adv. Join. Process..

[19] Tandon V., Thombre M.A., Patil A.P., Taiwade R.V., Vashishtha H. (2020). Effect of Heat Input on the Microstructural, Mechanical, and Corrosion Properties of Dissimilar Weldment of Conventional Austenitic Stainless Steel and Low-Nickel Stainless Steel. Met. Microstruct. Anal..

[20] Vashishtha H., Taiwade R.V., Sharma S., Patil A.P. (2017). Effect of welding processes on microstructural and mechanical properties of dissimilar weldments between conventional austenitic and high nitrogen austenitic stainless steels. J. Manuf. Process..

[21] Ibrahim I.R., Khedr M., Mahmoud T.S., Abdel-Aleem H.A., Hamada A. (2021). Study on the Mechanical Performance of Dissimilar Butt Joints between Low Ni Medium-Mn and Ni-Cr Austenitic Stainless Steels Processed by Gas Tungsten Arc Welding. Metals.

[22] Hamada A., Ghosh S., Ali M., Jaskari M., Järvenpää A. (2022). Studying the strengthening mechanisms and mechanical properties of dissimilar laser-welded butt joints of medium-Mn stainless steel and automotive high-strength carbon steel. Mater. Sci. Eng. A.

[23] Jang A., Lee D., Lee S., Shim J., Kang S., Lee H. (2011). Effect of Cr/Ni equivalent ratio on ductility-dip cracking in AISI 316L weld metals. Mater. Des..

[24] Shankar V., Gill T.P.S., Mannan S.L., Sundaresan S. (2003). Solidification cracking in austenitic stainless steel welds. Sadhana.

[25] Lu B., Chen Z., Luo J., Patchett B., Xu Z. (2005). Pitting and stress corrosion cracking behavior in welded austenitic stainless steel. Electrochim. Acta.

[26] Nagasai B.P., Malarvizhi S., Balasubramanian V. (2022). Mechanical properties and microstructural characteristics of wire arc additive manufactured 308 L stainless steel cylindrical components made by gas metal arc and cold metal transfer arc welding processes. J. Mater. Process. Technol..

[27] Kshirsagar R., Jones S., Lawrence J., Tabor J. (2019). Measurement of ferrite content of stainless steel sheet welds using a new Ferrite Density Number scale. J. Mater. Process. Technol..

[28] Bhanu V., Gupta A., Pandey C. (2022). Role of A-TIG process in joining of martensitic and austenitic steels for ultra-supercritical power plants–A state of the art review. Nuclear Eng. Technol..

[29] Weman K. (2012). 1—Introduction to welding. Welding Processes Handbook.

[30] Oliver W.C., Pharr G.M. (1992). An improved technique for determining hardness and elastic modulus using load and displacement sensing indentation experiments. J. Mater. Res..

[31] (2021). Standard Test Methods for Tension Testing of Metallic Materials.

[32] Tian Y., Lin S., Ko J.P., Lienert U., Borgenstam A., Hedström P. (2018). Micromechanics and microstructure evolution during in situ uniaxial tensile loading of TRIP-assisted duplex stainless steels. Mater. Sci. Eng. A.

[33] Baghdadchi A., Hosseini V.A., Karlsson L. (2021). Identification and quantification of martensite in ferritic-austenitic stainless steels and welds. J. Mater. Res. Technol..

[34] Walentek A., Seefeldt M., Verlinden B., Aernoudt E., VAN Houtte P. (2006). Electron backscatter diffraction on pearlite structures in steel. J. Microsc..

[35] Hietala M., Ali M., Khosravifard A., Keskitalo M., Järvenpää A., Hamada A. (2021). Optimization of the tensile-shear strength of laser-welded lap joints of ultra-high strength abrasion resistance steel. J. Mater. Res. Technol..

[36] Sabzi M., Anijdan S.M., Chalandar A.B., Park N., Jafarian H., Eivani A. (2022). An experimental investigation on the effect of gas tungsten arc welding current modes upon the microstructure, mechanical, and fractography properties of welded joints of two grades of AISI 316L and AISI310S alloy metal sheets. Mater. Sci. Eng. A.

[37] Khedr M., Wei L., Na M., Yu L., Xuejun J. (2019). Evolution of Fracture Mode in Nano-twinned Fe-1.1C-12.5Mn Steel. Jom.

[38] Drozdenko D., Bohlen J., Yi S., Minárik P., Chmelík F., Dobroň P. (2016). Investigating a twinning–detwinning process in wrought Mg alloys by the acoustic emission technique. Acta Mater..

[39] Kurz W., Bezençon C., Gäumann M. (2001). Columnar to equiaxed transition in solidification processing. Sci. Technol. Adv. Mater..

[40] Dak G., Pandey S.M., Pandey C. (2022). Residual stress analysis, microstructural characterization, and mechanical properties of tungsten inert gas-welded P92/AISI 304L dissimilar steel joints. Proc. Inst. Mech. Eng. Part L J. Mater.: Des. Appl..

[41] Teng T.-L., Chang P.-H., Tseng W.-C. (2003). Effect of welding sequences on residual stresses. Comput. Struct..

[42] Chuaiphan W., Somrerk C.A., Niltawach S., Sornil B. (2012). Dissimilar Welding between AISI 304 Stainless Steel and AISI 1020 Carbon Steel Plates. Appl. Mech. Mater..

[43] Khidhir G.I., Baban S.A. (2019). Efficiency of dissimilar friction welded 1045 medium carbon steel and 316L austenitic stainless steel joints. J. Mater. Res. Technol..

[44] Newbury D.E., Ritchie N.W.M. (2014). Performing elemental microanalysis with high accuracy and high precision by scanning electron microscopy/silicon drift detector energy-dispersive X-ray spectrometry (SEM/SDD-EDS). J. Mater. Sci..

[45] Newbury D.E., Ritchie N.W.M. (2012). Is Scanning Electron Microscopy/Energy Dispersive X-ray Spectrometry (SEM/EDS) Quantitative?. Scanning.

[46] El-Bitar T., El-Meligy M., Khedr M. (2020). Investigation of exhaust valve failure in a marine diesel engine. Eng. Fail. Anal..

[47] Uranga P., Shang C.-J., Senuma T., Yang J.-R., Guo A.-M., Mohrbacher H. (2020). Molybdenum alloying in high-performance flat-rolled steel grades. Adv. Manuf..

[48] Abioye T., Ariwoola O.E., Ogedengbe T., Farayibi P.K., Gbadeyan O. (2019). Effects of Welding Speed on the Microstructure and Corrosion Behavior of Dissimilar Gas Metal Arc Weld Joints of AISI 304 Stainless Steel and Low Carbon Steel. Mater. Today: Proc..

[49] Kolubaev A., Sizova O., Fortuna S., Vorontsov A., Ivanov A., Kolubaev E. (2020). Weld structure of low-carbon structural steel formed by ultrasonic-assisted laser welding. J. Constr. Steel Res..

[50] Gajjar P.K., Khatri B.C., Siddhpura A.M., Siddhpura M.A. (2022). Sensitization and Desensitization (Healing) in Austenitic Stainless Steel: A Critical Review. Trans. Indian Inst. Met..

[51] Doerr C., Kim J.-Y., Singh P., Wall J.J., Jacobs L.J. (2017). Evaluation of sensitization in stainless steel 304 and 304L using nonlinear Rayleigh waves. NDT E Int..

[52] Zhao B., Dai Q., Zhao Z.J., He M., Wang L.X., Zhang G.H. (2022). Study on Grain Boundary Sensitization in Heat Affected Zone of Austenitic Stainless Steel and its Evaluation Method. Mater. Sci. Forum.

[53] Devine T. (1990). The mechanism of sensitization of austenitic stainless steel. Corros. Sci..

[54] Maity J. (2022). An analytical model of grain growth considering the conjoint effects of precipitate pinning and solute drag in steel. Philos. Mag. Lett..

[55] Oikawa T., Zhang J.J., Enomoto M., Adachi Y. (2013). Influence of Carbide Particles on the Grain Growth of Ferrite in an Fe–0.1C–0.09V Alloy. ISIJ Int..

[56] Li Y., He Y., Liu J., Cui H., Qiu S., Zhang P., Zheng G. (2020). Phase transformation and microstructure evolution of pearlite heat-resistant steel during heating. Mater. Sci. Technol..

[57] Wang S., Wang Z., Zhang C., Wang Z. (2020). Numerical Simulation and Experimental Investigation on Electron Beam Welding of Spray-Formed 7055 Aluminum Alloy. Metals.

[58] Nikulina A., Smirnov A., Chevakinskaya A. (2014). Formation of a Transition Zone Structure in Welded Joints between Dissimilar Steels. Appl. Mech. Mater..

[59] Başyiğit A.B., Kurt A. (2018). The Effects of Nitrogen Gas on Microstructural and Mechanical Properties of TIG Welded S32205 Duplex Stainless Steel. Metals.

[60] Karimi A., Karimipour A., Akbari M., Razzaghi M.M., Ghahderijani M.J. (2023). Investigating the mechanical properties and fusion zone microstructure of dissimilar laser weld joint of duplex 2205 stainless steel and A516 carbon steel. Opt. Laser Technol..

